# Zinc and Diabetic Retinopathy

**DOI:** 10.1155/2013/425854

**Published:** 2013-03-17

**Authors:** Xiao Miao, Weixia Sun, Lining Miao, Yaowen Fu, Yonggang Wang, Guanfang Su, Quan Liu

**Affiliations:** ^1^The Second Hospital of Jilin University, Changchun 130021, China; ^2^Department of Ophthalmology, The Second Hospital of Jilin University, 218 Ziqiang Street, Changchun 130041, China; ^3^The First Hospital of Jilin University, Changchun 130021, China; ^4^Department of Cardiovascular Disease, The First Hospital of Jilin University, Changchun, Jilin 130021, China

## Abstract

Zinc (Zn) is an important nutrient that is involved in various physiological metabolisms. Zn dyshomeostasis is often associated with various pathogeneses of chronic diseases, such as metabolic syndrome, diabetes, and related complications. Zn is present in ocular tissue in high concentrations, particularly in the retina and choroid. Zn deficiencies have been shown to affect ocular development, cataracts, age-related macular degeneration, and even diabetic retinopathy. However, the mechanism by which Zn deficiency increases the prevalence of diabetic retinopathy remains unclear. In addition, due to the negative effect of Zn deficiency on the eye, Zn supplementation should prevent diabetic retinopathy; however, limited available data do not always support this notion. Therefore, the goal of this paper was to summarize these pieces of available information regarding Zn prevention of diabetic retinopathy. Current theories and possible mechanisms underlying the role of Zn in the eye-related diseases are discussed. The possible factors that affect the preventive effect of Zn supplementation on diabetic retinopathy were also discussed.

## 1. Introduction

Zinc (Zn) is the second most abundant trace element in the human body and is an important nutrient and cofactor of numerous enzymes and transcription factors [1–3]. There are more than 300 catalytically active Zn metalloproteinase and more than 2000 Zn-dependent transcription factors. Zn is involved in homeostasis, immune responses, oxidative stress, apoptosis, and aging. Zn homeostasis results from the coordinated regulation by metallothioneins (MTs) and proteins in the Zrt/Irt-like protein (ZIP) and Zn transporter (ZnT) families [4–7]. These proteins are involved in the uptake, excretion, and intracellular storage/trafficking of Zn. Abnormalities in Zn homeostasis, such as its deficiency, may be associated with various pathogeneses of chronic diseases.

Metallothionein (MT) play a key role in scavenging of free radicals and is the main regulator of the intracellular transport and mobilization, storage, and transferring of Zn [8]. It is a cysteine-rich protein that binds metals such as Zn and copper and acts as an antioxidant that is very efficient in scavenging various free radicals or reactive oxygen species (ROS) [9, 10].

Diabetes mellitus affects 200 million people worldwide [11], including 20 million people in the United States alone [12]. Diabetic retinopathy (DR), a specific microvascular complication of diabetes, is the leading cause of blindness in working-aged persons in the United States [12]. The prevalence of DR increases with duration of diabetes [13], and nearly all persons with type 1 diabetes and more than 60% of those with type 2 have some retinopathy after 20 years.

Clinically, DR can be classified as nonproliferative DR (NPDR) and proliferative DR (PDR) [14]. NPDR is characterized ophthalmoscopically by the presence of microaneurysms and dot and blot hemorrhages. Severe NPDR (also called preproliferative DR) shows increased retinal microvascular damage as evidenced by cotton wool spots, venous beading, venous loops, and intraretinal microvascular abnormalities. If left untreated, PDR (characterized by abnormal retinal neovascularization) can develop. Clinically important outcomes of PDR are retinal and vitreous hemorrhage and tractional retinal detachment [14], which ultimately result in blindness. Previous studies have shown that Zn supplementation attenuates oxidative changes at the early stage of diabetic rats, potentially preventing the early stages of DR, and delay its progression.

Several complications of diabetes may be related to increased intracellular oxidants and free radicals associated to decreases in intracellular Zn and Zn-dependent antioxidant enzymes [15]. Zinc effectively ameliorates diabetes-related complications in various animal models [16]. It is also an effective inducer of gene and protein expressions of MT, a potent antioxidant [17]. Therefore, the goal of the present paper was to summarize the information from the literature regarding (1) the role of Zn in diabetes, (2) the effect of Zn on the eye, and (3) the evidence that Zn prevents DR, as well as the possible mechanisms underlying this role. Finally, the future of Zn therapy for DR is briefly discussed.

## 2. Zinc and Oxidative Stress

It is well accepted that oxidative stress is elevated in various tissues that are associated with microvascular and macrovascular complications of diabetes [18, 19]. Increased oxidative stress contributes to the development of DR [20, 21], as an increase in reactive oxygen species (ROS) is considered a causal link between elevated glucose and other metabolic abnormalities that are important in its development [22]. Various antioxidants and nutrients have provided encouraging results in experimental models of DR [20, 21], although the results from clinical trials have been less conclusive [23–25]. In diabetic mice, overexpression of manganese superoxide dismutase (MnSOD), the enzyme responsible for scavenging mitochondrial superoxide, prevents early retinal lesions of retinopathy [26].

**Figure 1:**
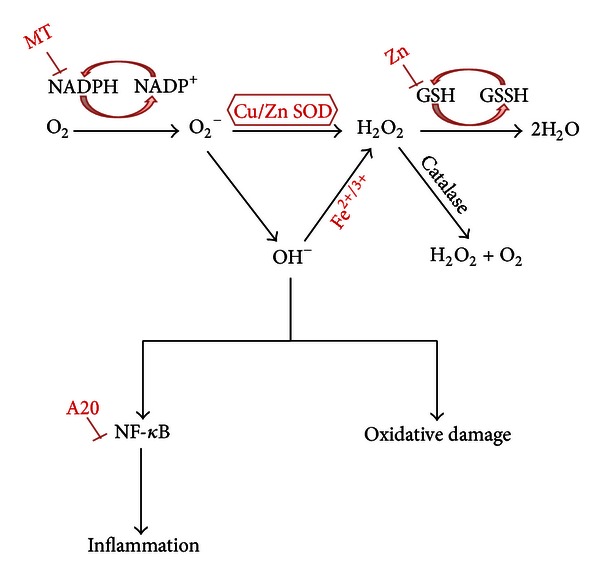
Proposed mechanism of zinc effect on oxidative stress and inflammation. Zinc attenuates oxidative damage and inflammation via MT, Cu/Zn SOD, Zn-finger protein, and itself.

Zn has antioxidant properties and protects tissue from oxidative stress by two main mechanisms: (i) protection of protein sulfhydryl groups from free radical attack and (ii) reduction of free radical formation through the antagonism of redox-active transition metals, such as iron and copper (Cu) [27]. Each of these mechanisms results in decreased reactivity of sulfhydryl groups. The protection of protein sulfhydryls is thought to involve reduction of sulfhydryl reactivity through one of three mechanisms: (i) direct binding of Zn to sulfhydryl groups, (ii) steric hindrance as a result of Zn binding to another protein site in close proximity to the sulphydryl group, and (iii) a conformational change that results from Zn binding to another site on the protein. Some examples of proteins that Zn protects are dihydroorotase, DNA Zn-binding proteins (Zn fingers), and protein farnesyltransferase (Figure 1) [28].

As an antioxidant, Zn reduces the formation of free radicals by acting as an inhibitor of NADPH oxidase, which is an inducer of MTs (free radical scavengers) and an integral metal of Cu/Zn-SOD. ROS are known to activate NF-*κ*B, which in turn, activates growth factors, antiapoptotic molecules resulting in cell proliferation (cancer), inflammatory cytokines, and adhesion molecules [29]. Zn also reduces inflammatory cytokine production by upregulating a Zn-finger protein, A20, which inhibits NF-*κ*B activation via the TRAF pathway [30]. Thus, Zn not only functions as an antioxidant, but also as an anti-inflammatory agent (Figure 1). In contrast to plasma Cu, plasma Zn concentrations and the Zn/Cu ratio were lower in diabetic subjects [31]. Meanwhile, it has been suggested that Zn supplementation protects against oxidative changes in the early stages of diabetes [32]. Zn supplementation also appears to have beneficial antioxidant effects in people with type 2 diabetes [33] (Figure 1).

## 3. Zinc and the Eye

Zn is indispensable to the growth and development of microorganisms, plants, and animals. It is found in all body tissues and secretions in relatively high concentrations, with 85% of whole body Zn found within the muscle and bones, 11% found in the skin and the liver, and the remaining found in all other tissues, with the highest concentrations in the prostate and parts of the eye [34], such as retina [35]. Zn appears to play an integral role in maintaining normal ocular function and is present in high concentrations in the ocular tissue, particularly in the retina and the choroid. Zn deficiency has been shown in a number of species to result in a variety of gross, ultrastructural, and electrophysiologic ocular manifestations.

Zn deficiency in rats dramatically affects ocular development. Severe Zn deficiency, when imposed upon rats during gestation, results in optic cup invagination failure, colobomata, retinal dysplasia, and occasionally anophthalmia in pups [36]. Acrodermatitis enteropathica is a rare early childhood disease, with multiple systemic manifestations caused by abnormalities in Zn metabolism. The ocular abnormalities include blepharitis, photophobia, conjunctivitis, corneal opacities, and cataracts [37]. Superficial punctate opacities, nebulous subepithelial opacities, and linear corneal erosions have also been reported in cases of acrodermatitis enteropathica [37]. Recently, differential display was used to investigate gene expression in acrodermatitis enteropathica, and the results showed an insertional mutation that affects the mRNA of a Zn transport protein, resulting in decreased Zn absorption [38]. Recalcitrant corneal ulcers have been reported in conjunction with low serum Zn [39]. In addition, Zn has been indirectly linked with corneal ulcers because it may be required for the functional activity of collagenases [40].

Zn deficiency in humans results in poor dark adaptation and night blindness [41, 42], which in most cases can be reversed by Zn supplementation [43]. These alterations appear to be the consequence of defects in retinol processing in retinal pigment epithelial (RPE) cells during the visual cycle. The Zn metalloenzyme, retinol dehydrogenase, catalyzes the oxidation of retinol to retinal, and animal experiments and *in vitro* studies have demonstrated that the activity of retinol dehydrogenase can be impaired by Zn deficiency [44]. The above-mentioned changes in the localization of histochemically reactive Zn within the dark- and light-adapted states of the photoreceptor suggest that Zn possesses a unique role in the phototransduction process and/or the photoreceptor-RPE interaction, as occurs for vitamin A [45]. The presence of reactive Zn (Zn^2+^) within photoreceptor terminals, and the evidence that exogenous Zn affects the electrophysiological activity of the distal retina, has led to the presumption that its corelease with glutamate may play an essential role in the modulation of information at the first synapse in the visual pathway. Although Zn release can be visualized in the outer synaptic layer of a retinal slice preparation, it cannot be ascertained with certainty that the release sites are at the presynaptic terminal and not from the mitochondria-rich inner segment or within the distal processes of photoreceptors and Müller cells. Synaptically released Zn may significantly influence neural processing in the vertebrate retina by modulating the activity of excitatory and/or inhibitory receptors as well as intracellular signaling proteins [46]. Therefore, the possibility exists that abnormal dark adaptation in Zn-deficient states is the result of an impaired Zn-dependent reaction in the visual cycle within the photoreceptors.

Cataracts are a common disease in older adults worldwide. Although some epidemiological studies have shown Zn involvement in the development of cataracts, the lowest concentration of Zn in crystalline lenses has been detected in patients with mature senile cataracts, while the highest concentrations have been detected in patients with traumatic cataracts [47]. However, the results from the largest randomized trial done in the United States showed no beneficial effect of supplement Zn with cupric oxide on the development or progression of cataracts [48]. 

Zn has a strong effect on the acceleration of MT synthesis [49]. Some studies have reported that MT-III plays a pivotal role as an endogenous neuroprotectant against light-induced retinal damage [50]. The mRNAs of MT isoforms (I–III) were upregulated in the murine retina by light exposure, and light-induced retinal damage is exacerbated in MT-III-deficient mice [50]. Furthermore, it has also been demonstrated that MT isoforms I and II do not have pivotal roles in protecting against light-induced retinal photoreceptor cell loss, whereas MT-III has neuroprotective effects, possibly due to its strong interaction with ROS.

Age-related macular degeneration is a leading cause of visual loss in older adults and is characterized by accumulation of membranous debris on both sides of the RPE basement membrane. This condition is thought to result from oxidative stress, and Zn deficiency is involved in its pathogenesis. It has been demonstrated that Zn [51, 52] and MT [53] levels are reduced in the RPE of the human eye in aged-related macular degeneration. In accordance with this, a study using a monkey model of early-onset aged-related macular degeneration [54] showed a four-fold decrease in retinal Zn content, decreased synthesis of MT, and increased oxidative stress in affected retinas compared to unaffected controls. In addition, it has been suggested that Zn supplementation prevents the appearance of age-related macular degeneration [55] and decreases the progression of the dry form of the disease [56]. These findings support the view that Zn deficiency is involved in the pathogenesis of age-related macular degeneration. Moreover, a clinical study showed a significant reduction in the Zn/Cu ratio in serum involved in the development of DR, thus demonstrating the importance of Zn in disease progression [57].

## 4. Zn Plays an Important Role in DR

Zn is a key element for maintenance of the structural and functional integrity of eukaryotic cells and tissues [58]. Many studies have addressed the importance of Zn as an antioxidant and therapeutic agent in several free radical initiation systems [59–61]. Some studies have reported the beneficial effects of antioxidants and Zn supplementation in preventing progression to advanced age-related macular degeneration, and people supplemented with antioxidants and Zn are less likely to lose visual acuity [62]. Moreover, Zn has been shown to protect the retina from diabetes-induced increased lipid peroxidation and decreased glutathione levels in rats either by stabilizing the membrane structure or by inducing MT synthesis [32]. Zn is essential for Cu-Zn SOD and inhibits diabetes-induced increases in plasma malondialdehyde and decreases in erythrocyte antioxidant defense enzymes [63]. Cu oxide functions as the active center of many cuproenzymes, including Cu-Zn SOD [64], and Cu deficiencies result in oxidative damage to lipids, DNA, and proteins [65]. The precise mechanism by which Zn and Cu exert their protective effects against retinal damage remains unclear, but there is a strong possibility that these nutrients help decrease oxidative damage.

It is apparent that DR can best be managed by tight glycemic control [21]. Moreover, antioxidant therapy may be a suitable approach for inhibiting intrinsic changes within the retinal capillary bed that leads to the development of DR. A previous study showed that alloxan causes a reduction in GSH levels and an increase in the levels of lipid peroxidation products (TBA reactants) in the retinas of diabetic rats, supporting the role of oxidative stress in the development of DR [32]. Many studies have addressed the importance of antioxidants in the control of abnormalities in diabetic retinas [66–68]; however, many of these studies have indicated the inability of these antioxidants to lower blood hexose levels [20, 67, 69, 70]. Other studies have indicated the inability of some antioxidants to inhibit lipid peroxidation in diabetic eyes [71]. Above all, these studies reveal the ability of Zn to both minimize perturbations in plasma glucose levels in alloxan-diabetic rats and ameliorate deteriorative changes in the levels of TBA reactants and GSH in the retina [32].

The previous study indicates increased lipid peroxidation in the retinas of alloxan-treated rats that was associated with increased plasma glucose levels. On the other hand, the study reported elevated rates of liver lipid peroxidation accompanied by deterioration in glucose tolerance in GSH-depleted rats [72]. It has been suggested that in free radicals initiation systems, deterioration in glucose tolerance is attributed to impaired insulin action [73]. One study showed that impaired insulin-stimulated glucose transport across the cell membrane is a major mechanism underlying age-associated glucose intolerance in aged rats [74]. Free radicals are hypothesized to be one of the underlying causes of aging [75]. Initiating lipid peroxidation by free radicals in the lipid moiety of the cell membrane is presumed to result in distortion of the structural and functional integrity of the cell membrane or internal cellular components. This would interfere with the ability of insulin to initiate and propagate its normal sequence of actions [74], which may account, at least in part, for alloxan-induced hyperglycemia. Previous studies have shown that the treatment of alloxan-diabetic rats with Zn chloride resulted in the reduction of both plasma glucose levels and lipid peroxidation in the retinas of these rats [32]. The protective effects of Zn against increases in lipid peroxidation may be due to its ability to bind and stabilize cellular membranes against lipid peroxidation and disintegration [76]. An alternative protective mechanism of Zn may be its ability to induce MT synthesis. The high sulfhydryl content enables MT to efficiently scavenge oxyradicals [77, 78]. Another possible protective mechanism of MT is its ability to release Zn to bind to sites on membrane surfaces, thereby displacing adventitious iron and inhibiting lipid peroxidation [76]. Moreover, the suggested effect of Zn in inducing SH-rich MT synthesis may preserve the SH residue in many functional proteins. Therefore, Zn may preserve the structural and functional integrity of SH-dependent enzymes, including those that regulate glucose metabolism. More recently, it was hypothesized that MT, which is cysteine-rich, plays a role in nitric oxide signaling events via sequestration or release of Zn^2+^ by the unique thiolate clusters of the protein [79].

The protective effect of Zn against lipid peroxidation that is observed in the retinas of alloxan-diabetic rats could be of considerable importance for halting the progression of diabetes-related retinal degeneration, since a strong positive correlation has been found between lipid peroxidation products and vascular endothelial growth factor (VEGF) concentrations in the vitreous of patients with proliferative DR [68]. Moreover, one type of SOD, a major antioxidant enzyme, is Zn-dependent (Cu/Zn-SOD). Cu/Zn-SOD is a potent antioxidant enzyme that has recently been proposed to have a tumor-suppression effect [80]; therefore, its role in the protection against the development of DR should not be excluded. Thus, the importance of Zn as a protective antioxidant against DR may lie in its ability to initially exert good glycemic control, thus inhibiting the development of the deleterious consequences of hyperglycemia. In addition, Zn may be an important factor in inhibiting the progression of the intrinsic changes in diabetic retinas that eventually lead to the development of DR.

## 5. Possible Mechanisms of Zn Prevention of DR

As discussed above, Zn acts as a potent antioxidant. Therefore, the antioxidant action of Zn could be considered as the first possible mechanism. It is well known that hyperglycemia accelerates the formation of advanced glycation end products (AGEs), which have been implicated in the pathogenesis of DR [81]. They can stimulate ROS production in retinal pericytes, largely via activation of NADPH oxidase, which results in retinal pericyte apoptosis [82]. Other studies have suggested that the critical role of oxidative stress in pericyte apoptosis, as treatment of diabetic rats with a mixture of different antioxidants [20] or with trolox [66], was able to prevent pericyte loss in retina. As discussed above, Zn can prevent cells from oxidative damage, and NADPH oxidase is inhibited by zinc and SOD, which is both a zinc and copper-containing enzyme [29]. It is suggested that Zn might prevent retinal pericyte apoptosis via inhibition of NADPH oxidase in DR.

**Figure 2:**
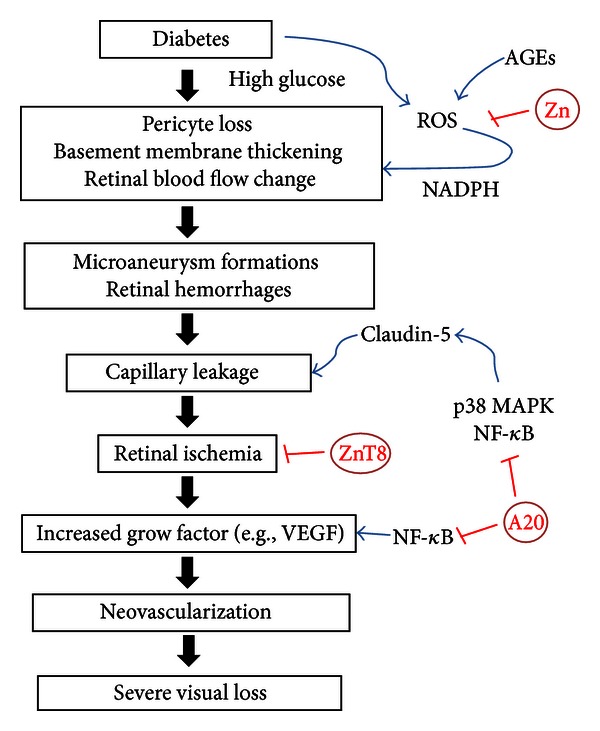
Proposed mechanism by which zinc protects from DR. Zinc protects DR by suppressing the pericyte apoptosis, capillary leakage, and neovascularization.

Ocular neovascularization, which is most potently caused by hypoxia and ischemia, is also a key component in DR [83, 84]. It has been convincingly demonstrated that hypoxia inducible factor-1 (HIF-1) and VEGF are involved in the initiation and progression of neovascularization in DR [85]. This has led to the finding of many new agents targeting VEGF [86]. Ischemia and hypoxia are similar conditions in cancer. Zn reduces inflammatory cytokine production by upregulating the Zn-finger protein, A20, which inhibits NF-*κ*B activation via the TRAF pathway [30]. Some studies have shown that Zn supplementation can reduce VEGF expression by inhibiting NF-*κ*B in prostate cancer [87]. It has been suggested that Zn might prevent neovascularization by inhibiting VEGF expression in DR (Figure 2). In addition, recent finding suggests that ZnT8 expression was reduced by ischemic insults and to restore the ZnT8 to its basal homeostatic levels can prevent retinas from ischemia induced injury [88]. Ischemia is also a key component in DR. Therefore, Zn supplement might rescue retina from DR (Figure 2).

Vascular leakage is also an important part of DR. p38 MAPK and NF-*κ*B signaling pathways contribute to the regulation of claudin-5 expression, and these factors induce endothelial permeability [89]. It has been reported that Zn associates with p38 MAPK activation in the diabetic testis [90]. Moreover, recent studies have shown VEGF-induced vascular leakage in DR [91]. As discussed above, Zn might reduce VEGF by inhibiting NF-*κ*B under conditions of hypoxia and ischemia; thus Zn might prevent vascular leakage in DR (Figure 2).

## 6. Conclusions

Increased oxidative stress plays an important role in many human diseases, such as diabetes and its complication. Zn supplementation seems beneficial for the patients with diabetes to control glucose levels. Zn as an antioxidant or via induction of MT attenuates ROS effect. Zn might protect retina from ROS induced pericytes apoptosis, capillary leakage, and neovascularization (Figure 2), thereby might have protective on DR. However whether this means that Zn supplementation can immediately be used to treat or prevent DR remains to be determined. A promising future for Zn supplementation will warrant further studies.

## Abbreviations

AGEs: Advancedglycationendproducts
Cu: Copper
DR: Diabeticretinopathy
Znfingers: DNAZn-bindingproteins
HIF-1: Hypoxiainduciblefactor-1
MT: Metallothionein
MnSOD: Manganesesuperoxidedismutase
NF-*κ*B: NuclearfactorkB
NPDR: NonproliferativeDR
PDR: ProliferativeDR
RPE: Retinalpigmentepithelialcells
ROS: Reactiveoxygenspecies
VEGF: Vascularendothelialgrowthfactor
Zn: Zinc
ZIP: Zrt/Irt-likeprotein
ZnT: Zntransporter.
